# Integrating morphology and metagenomics to understand taxonomic variability of *Amphisorus* (Foraminifera, Miliolida) from Western Australia and Indonesia

**DOI:** 10.1371/journal.pone.0244616

**Published:** 2021-01-04

**Authors:** Jan-Niklas Macher, Martina Prazeres, Sarah Taudien, Jamaluddin Jompa, Aleksey Sadekov, Willem Renema

**Affiliations:** 1 Naturalis Biodiversity Center, Leiden, The Netherlands; 2 Helmholtz-Institut für Funktionelle Marine Biodiversität (HIFMB), Oldenburg, Germany; 3 Hasanuddin University, Makassar, Indonesia; 4 ARC Centre of Excellence for Coral Reef Studies, Ocean Graduate School, The University of Western Australia, Crawley, Australia; 5 Department of Ecosystem & Landscape Dynamics, Institute for Biodiversity & Ecosystem Dynamics (IBED), University of Amsterdam, Amsterdam, The Netherlands; Universita degli Studi di Urbino Carlo Bo, ITALY

## Abstract

Foraminifera are a group of mostly marine protists with high taxonomic diversity. Species identification is often complex, as both morphological and molecular approaches can be challenging due to a lack of unique characters and reference sequences. An integrative approach combining state of the art morphological and molecular tools is therefore promising. In this study, we analysed large benthic Foraminifera of the genus *Amphisorus* from Western Australia and Indonesia. Based on previous findings on high morphological variability observed in the Soritidae and the discontinuous distribution of *Amphisorus* along the coast of western Australia, we expected to find multiple morphologically and genetically unique *Amphisorus* types. In order to gain detailed insights into the diversity of *Amphisorus*, we applied micro CT scanning and shotgun metagenomic sequencing. We identified four distinct morphotypes of *Amphisorus*, two each in Australia and Indonesia, and showed that each morphotype is a distinct genotype. Furthermore, metagenomics revealed the presence of three dinoflagellate symbiont clades. The most common symbiont was *Fugacium* Fr5, and we could show that its genotypes were mostly specific to *Amphisorus* morphotypes. Finally, we assembled the microbial taxa associated with the two Western Australian morphotypes, and analysed their microbial community composition. Even though each *Amphisorus* morphotype harboured distinct bacterial communities, sampling location had a stronger influence on bacterial community composition, and we infer that the prokaryotic community is primarily shaped by the microhabitat rather than host identity. The integrated approach combining analyses of host morphology and genetics, dinoflagellate symbionts, and associated microbes leads to the conclusion that we identified distinct, yet undescribed taxa of *Amphisorus*. We argue that the combination of morphological and molecular methods provides unprecedented insights into the diversity of foraminifera, which paves the way for a deeper understanding of their biodiversity, and facilitates future taxonomic and ecological work.

## Introduction

Species identification in foraminifera, a diverse group of mostly marine protists, is often complex, and so open questions regarding their species diversity, biogeography, and genetic diversity remain [1–3]. Gaining a more profound understanding of foraminiferal diversity is needed for reliable species identification and description. While morphological and molecular studies on foraminifera have led to the discovery of new species [2, 4–7], they also underpinned that species identification can be challenging due to incongruences of molecular and morphological evidence [8]. Molecular approaches routinely report a high number of previously unrecognized foraminiferal taxa [9, 10], but some (e.g., *Uvigerina*) show high morphological plasticity which is not reflected in genetic variability [11]. Others (e.g. *Ammonia*) show a high genetic variability which is not reflected in their morphology [12]. To address these issues, using an integrated approach is beneficial, as shown by previous studies [5, 13].

In this study we assess the morphological and molecular diversity of the genus *Amphisorus* (Soritidae, Miliolida) from Western Australia. *Amphisorus* is part of the informal grouping commonly referred to as large benthic foraminifera (LBF), which live in obligate association with microalgal symbionts [14, 15] that are potentially host-specific [16]. Furthermore, they have a diverse prokaryotic microbiome [17, 18] which includes potentially symbiotic taxa [1]. To gain more detailed insights into the diversity of *Amphisorus*, we enhance the integrative morphological and molecular approach by studying the genetic diversity of the algal symbionts, and assess the community composition of associated bacteria. Until recently, the widespread genus *Amphisorus* was regarded as monospecific, but at least three species have been formally described based on their morphology [19, 20], and additional phylotypes were recognised [16]. Based on the high morphological variability observed in the Soritidae [8], and the discontinuous distribution of *Amphisorus* along the coast of western Australia, we expected to find multiple morphologically and genetically unique types of *Amphisorus* along a north-south transect running 800km from Bush Bay to Rottnest Island (Western Australia). We expected the dinoflagellate symbionts of *Amphisorus* to show host-specificity, and we further tested whether *Amphisorus* morphotypes are associated with distinct bacterial communities. We argue that a combination of morphological methods and shotgun metagenomic approaches allow yet unprecedented insights into the diversity of foraminifera, their symbionts and associated bacteria, thereby facilitating the understanding of foraminifera biodiversity.

## Material and methods

### Ethics statements

Sampling and work on Foraminifera did not require ethical approval from authorities, as Foraminifera are protists. Sampling permissions were issued to Willem Renema for Indonesia (Ristek, No. 1497021734) and to Aleksey Sadekov for Australia (WA, Dept. of Parks and Wildlife, Rec No.SW019230 and 08-001845-1).

### Sampling

*Amphisorus* specimens were collected from five sites along the Western coast of Australia in September 2018 (Coordinates: S1 Table in S3 File). Further, *Amphisorus* specimens from Southwest Sulawesi (Spermonde archipelago, Indonesia, collected in April-May 2018) were used for morphological and genetic comparison to the Australian *Amphisorus* specimens. All specimens were collected from seagrass or sediment and morphologically identified as *Amphisorus* in the field (photos of specimens in their habitat: S1 Fig). Specimens were immediately stored in 96% ethanol and transported to Naturalis Biodiversity Center (Leiden, Netherlands) for further morphological and molecular analyses.

### Morphotype identification

**Fig 1:**
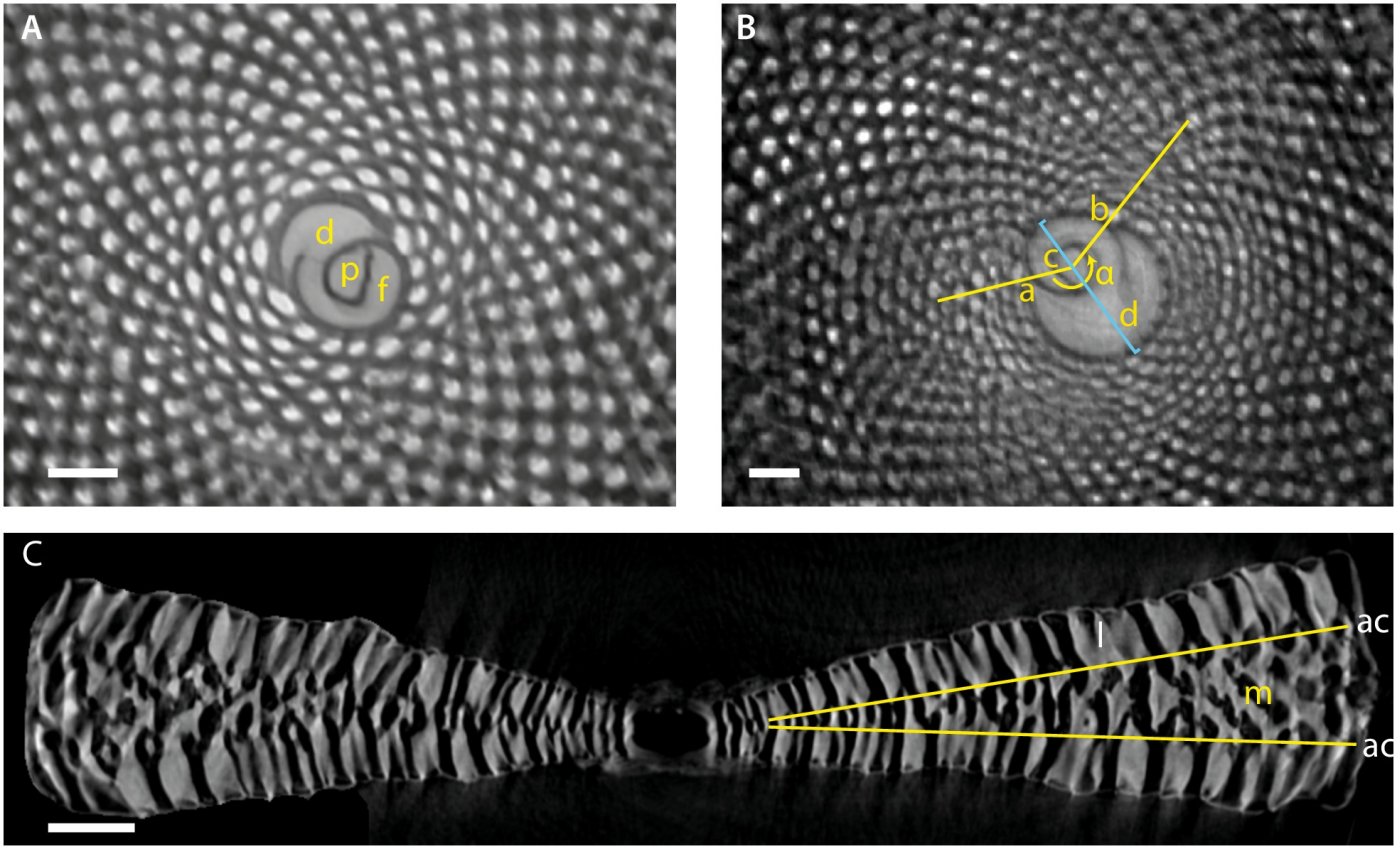
A) Terminology used in this paper p: Proloculus; f: Flexostyle; d: Deuteroconch; B) Explanation of the biometric methods used in the paper. α is the angle between the lines connecting the proximal intersect of the deuteroconch with the flexostyl (a), the center of the protoconch (c) and the lateral intersect of the deuteroconch with the flexostyle (b). The diameter of the embryonic apparatus is the maximum diameter of the protoconch+ deuteroconch+flexostyle (d). C) Terminology used in the axial section: l: Lateral chamberlets; m: Median skeleton or marginoporid structure (stolons of this structure form the median apartures on the apertural face); ac: The plane formed by the annular stolons of each chamberlet. These end in the median apertures on the apetural face. Scale bar = 100 μm.

Specimens were separated and carefully cleaned with sterile water to remove biofilm and sediment particles adherent to the test. Only macrospheric specimens were included in the morphological analysis, since the embryonic aparatus of microspheric forms were too thin walled to make reliable biometrical measurements. In total 88 macrospheric foraminifera were scanned using a Skyscan 1172 microCT (Bruker, Billerica, Massachusetts, USA) at 1–3 μm voxel size. Settings used for scanning were: binning 4000 x 2672 pixels; no filter; 60 kv, camera rotation steps of 0.1–0.25 degrees, and an exposure time of 700–1100 ms, depending on the size of the specimen and the resolution of the scan. Original X-ray projections were reconstructed using the NRecon software. Visualisations of the interseptal space were made using Avizo (v. 9.4). For each specimen, we measured embryon size and shape (see Fig 1A and 1B for a definition of terminology used in this paper), and checked for qualitative characters of the axial section (presence/absence of median skeleton, size of lateral chamberlets Fig 1C) of and the apertural face (shape of apertures, presence/absence of rim around apertures, presnence/absence of median apertures) as described in Gudmundsson [19]. These characteristics were used to determine the morphotype of the analysed specimens. To enhance the morphological dataset, specimens were further compared to dry specimens from Little Armstrong Bay (Rottnest Island, West Australia), which are stored in the Naturalis collection. The identified morphotypes were compared to published descriptions of *Amphisorus kudakajimaensis* [19], *A*. *sauronensis* [20], and *A*. *hemprichii* [21].

### Molecular analyses

The analysis of genetic diversity of host, symbiont, and bacterial associates was conducted using shotgun metagenomics. Therefore, total genomic DNA extraction was carried out using the QIAamp DNA Micro Kit (Qiagen; Hilden, Germany) from a subset of 38 fresh specimens preserved in 96% ethanol that could be morphologically identified as belonging to one of the four morphotypes present in our dataset (see S2 Table in S3 File). Prior to DNA extraction, specimens were photographed using a Zeiss Discovery v12 stereo microscope (Oberkochen, Germany).

*Amphisorus* specimens were individually dried in sterile petri dishes, added to 1.5-ml Eppendorf tubes containing extraction buffer, and broken into fine powder with sterile metal homogenisers. Further extraction procedure followed the manufacturer’s protocol, with the difference of proteinase K digestion for 12 hours over night to improve cell lysis. A negative control (sterile water) was processed together with the samples to check for potential contamination. After extraction, DNA quantification was conducted using the QIAxcel system (Qiagen; Hilden, Germany). Fragmentation of DNA was conducted using the Covaris M220 system (Brighton, Great Britain) using ultrasonication, with a target fragment size of 250 base pairs. Fragment quantity and length were checked on the QIAxcel. Shotgun metagenomic libraries were prepared using the New England Biolabs NEBNext Ultra II DNA Library Prep Kit (Ipswitch, USA) and the corresponding NEBNext Multiplex Oligos for Illumina, according to manufacturer’s protocol. Final concentration and fragment size were checked on the QIAxcel. Samples were equimolar pooled using the QIAcube system (Qiagen). No DNA was observed in the negative control, and it was added to the final library with 10% of the library volume. Fragment size and DNA concentration in the final library was checked on the Bioanalyzer system (Agilent Technologies, Santa Clara, USA) before sending for sequencing on the Illumina NovaSeq 6000 platform (2x150 bp read length) at Macrogen (Seoul, South Korea). Raw data was checked for low quality samples using the FastQC software [22]. Trimmomatic [23] with default settings was used to trim Illumina adapters. Trimmed reads were subject to a strict quality filtering using vsearch [24], with reads truncated at the first base with a phred score <15 using the ‘fastq-truncqual’ option to prevent including any low quality basepairs in subsequent genotyping analysis. All reads of less than 100bp length were discarded.

#### Assembly of *Amphisorus* and dinoflagellate genes

Geneious Prime (v. 2019.2, www.geneious.com) was used for assembly of the *Amphisorus* nuclear 18S rRNA—ITS1- 5.8S rRNA—ITS2—28S rRNA fragment. 18S rRNA is the commonly used barcoding gene for Foraminifera [25]. *Amphisorus* 18S rRNA reads were identified by mapping each sample against an 18S rRNA sequence of *Amphisorus hemprichii* downloaded from Genbank (accession number: AJ842184.1 [16]). Mapping was performed using the Geneious mapper with minimum 100 bp overlap, 10bp word length and 3% mismatch allowed. To confirm the results of this approach, a test with up to 10% mismatch allowed was performed, but resulted in the same consensus. The obtained consensus sequence was extracted and subsequently used as a reference. The sequence was extended by repeatedly mapping quality filtered reads against the consensus using the Geneious mapper (Settings: 1% gaps allowed, maximum gap size 2bp, maximum 5% mismatch per read, word length 15) until no further reads could be mapped. The settings were chosen as genetic variability within Foraminifera specimens has been reported to reach up to 5% [26]. This procedure was repeated for each specimen. Since no references from the order Miliolida were available, the 5.8S rRNA and 28S rRNA of *Amphisorus* were identified using the Geneious annotation tool, with *Uvigerina peregrina* 5.8S (Genbank accession: AY914598.1 [11]) and *Pulleniatina obliquiloculata* 28S rRNA (Genbank accession: LC049330.1 [27]) as references. The obtained *Amphisorus* sequences were aligned using MAFFT v. 7.4 [28], gaps in the alignment were removed, and all sequences were cut to the same length to allow further analyses. The assembled *Amphisorus* sequences were compared to existing 18S rRNA references (V4 region) reported as *Amphisorus hemprichii* from Rottnest Island (Western Australia) [16], where the Parker Point sampling site is located, and other *Amphisorus* sequences available in GenBank (36 sequences spanning the 18S V4 region). Sequences from our study were MAFFT aligned with these references, and pairwise identity was assessed using Geneious.

To identify the endosymbiotic dinoflagellates, the quality filtered reads of each specimen were mapped against the 28S rRNA sequences of 76 Dinophyceae species recently used for a phylogeny and revision of the Symbiodiniaceae [14]. We chose this marker due to the availability of this extensive, recently published reference database which allowed species identification with high confidence. Mapping was conducted with a minimum of 100bp overlap, 10bp word length and maximum 3% mismatch allowed. 3% intraspecific distance was identified as an informative species level threshold by [14]. The identified 28S rRNA consensus sequences were extracted, aligned using MAFFT, and trimmed to the 606 bp fragment used in [14]. For analyses of genotypes of the dominant dinoflagellate *Fugacium* clade Fr5, sequences were only included in analyses if the full 28s rRNA fragment could be obtained with an average coverage of greater than 30.

For the visualisation of how genotypes of *Amphisorus* and the most common dinoflagellate (*Fugacium* clade Fr5 [14]) correspond to *Amphisorus* morphotypes, alignment positions with gaps were removed to account for potential indels [29], and alignments were imported into PopART [30]. Median- joining networks (as used for visualisation of genetic structure in closely related lineages or species [31]) were built to visualise genotypes, and how they correspond to the identified *Amphisorus* morphotypes. Nucleotide diversity π was calculated for the *Amphisorus* morphotypes West Australia Larger (WAL), West Australia Small (WAS) and Spermonde Large (SpL). Nucleotide diversity could not be calculated for morphotype SpS, as only one specimen was studied with molecular methods.

#### Microbial community analysis

The microbial community associated with *Amphisorus* specimens from Western Australia was identified using Anvi’io [32]. *Amphisorus* collected from Indonesia were not included in this analysis as only four specimens from two different morphotypes and two different sampling sites were available for molecular work. Quality filtered reads of all WAS and WAL specimens were subject to a co-assembly using megahit [33] with the ‘meta-large’ mode to account for large and complex metagenomes. Only contigs with >2000 bp length were retained. Prokaryotic and eukaryotic contigs were separated using EukRep [34]. Quality filtered reads of each sample were mapped back to the prokaryotic contigs using Bowtie2 [35] to gain information on the read number mapped to each contig. The hmms function (based on HMMER; http://hmmer.org/) implemented in Anvi’io was used to predict genes in the assembled contigs. All contigs were subsequently binned into metagenome-assembled genomes (MAG) [36] using Concoct [37], Maxbin2 [38] and Metabat2 [39]. All bins were manually checked and refined using the Anvi’io interactive visualisation. Finally, bins were filtered using DAS Tool [40], which removed bins with a completeness of less than 50% and a redundancy of more than 5%. Thereby we only retained and analysed MAGs that fulfilled at least the medium-quality draft genome standards outlined in [36]. All bioinformatic commands used can be found in S1 File. Taxonomic annotation of MAGs was performed using GTDB-Tk [41]. Community composition of identified MAGs was analysed using R (v. 3.5) and the ‘vegan’ [42] package based on relative abundance data as provided by Anvi’io. Skewness of the data was assessed using histograms, qqplots and the skewness function implemented in the R library ‘moments’ [43]. Since the data was highly skewed, we applied log-transformation using the decostand function in vegan. To test whether morphotype or sampling location had a stronger influence on microbial community composition, vegan was used to perform PERMANOVA analysis using the ‘adonis’ function, based on Bray-Curtis distances, with the *Amphisorus* morphotype (WAL, WAS) respectively the sampling site as predictor. The ‘metamds’ function implemented in vegan was used to calculate non-metric multidimensional scaling (NMDS) plots for visualisation of results (all scripts are available in S2 File).

## Results

### Morphological analyses of *Amphisorus*

**Fig 2:**
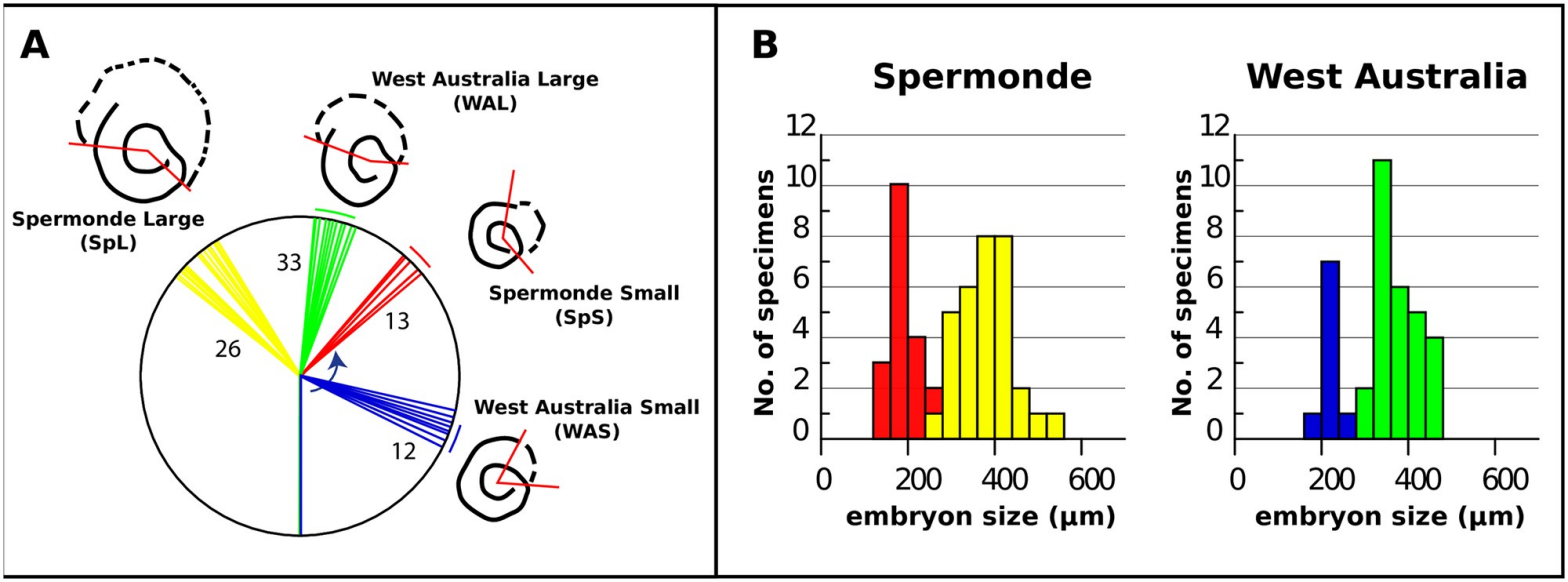
A) Variation in α (angle between the lines connecting the proximal intersect of the deuteroconch with the flexostyl) between the four *Amphisorus* morphotypes. Numbers indicate the number of analysed specimens B) Frequency diagram of the diameter of the embryonic apparatus (d in Fig 1) in Spermonde (A) and West Australia (B). Colors correspond to the morphotypes.

**Fig 3:**
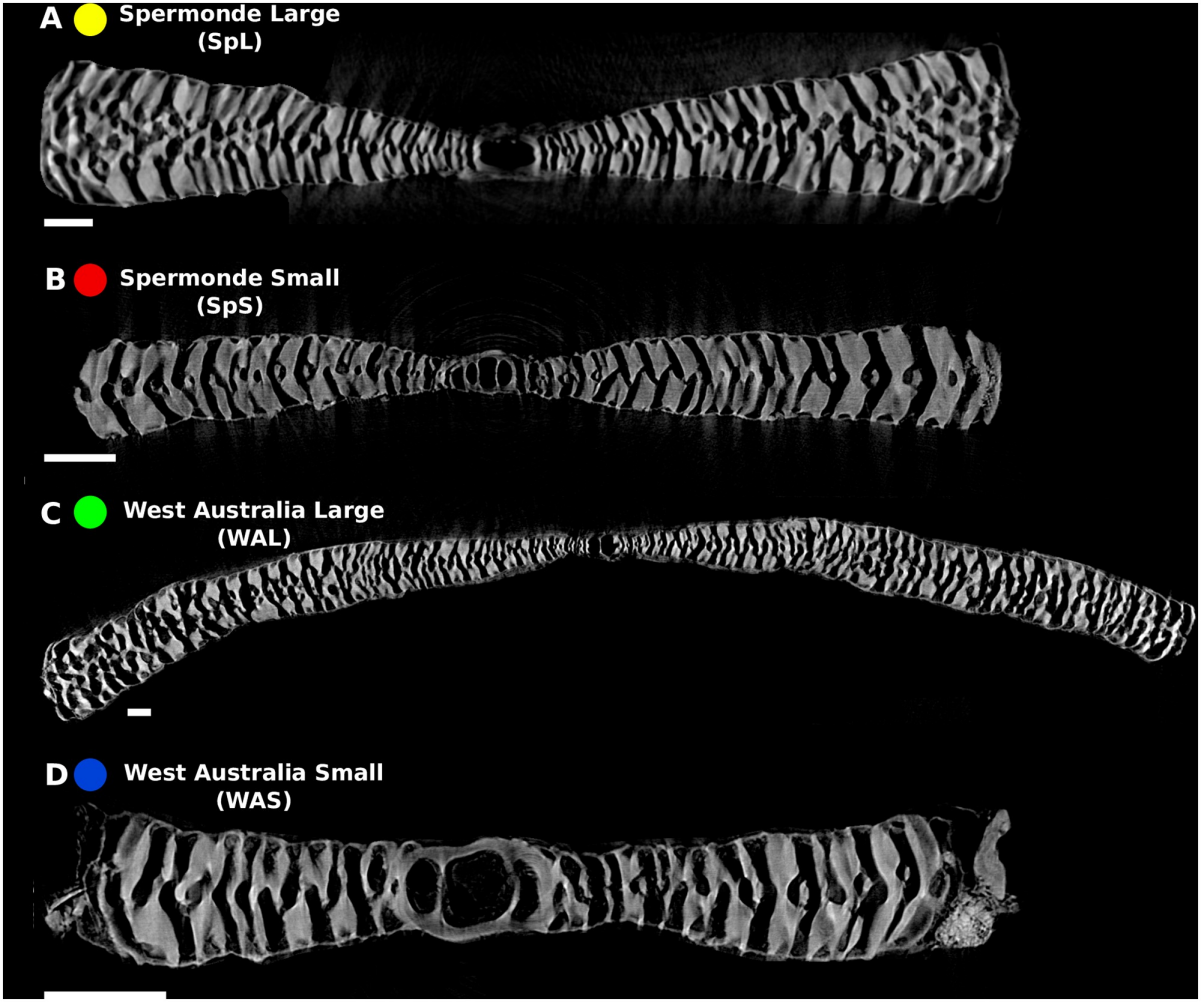
Virtual vertical cross sections through the A forms of the four morphotypes of *Amphisorus* recognised in this paper. A) Spermonde Large (SpL). Note the intermediate skeleton rapidly increasing in thickness towards the margin, while the marginal chamberlets are well developed and have a more or less constant thickness following the development of the intermediate skeleton. B) Spermonde Small (SpS). Note the absence of the intermediate skeleton. C) West Australia Large (WAL). Note the intermediate skeleton increasing in thickness towards the margin, while the marginal chamberlets are narroe and become thinner towards the margin. D) West Australia small (WAS). Note the absence of the intermediate skeleton. Scale bar = 200 μm.

Based on the combination of embryon size and shape, axial section, and the apertural face, we recognized four morphotypes (Figs 2 and 3 and S2 Fig). Detailed description and comparison of morphotypes are listed in Table 1. Two morphotypes were found in the main study region of Western Australia (Western Australia Small (WAS; 9 specimens); Western Australia Large (WAL; 28 specimens)), and two in the Spermonde region, SW Sulawesi, Indonesia (Spermonde Small (SpS; 19 specimens); Spermonde Large (SpL; 32 specimens)). Two morphotypes, one from Indonesia (SpL) and one from Australia (WAL), showed a large embryonic apparatus and a median skeleton. The two others had a smaller embryonic apparatus, again, one in the Spermonde (SpS), and one in Western Australia (WAS).

**Table 1:** Comparison of morphological characters of *Amphisorus* morphotypes described in this study (West Australia Large (WAL), West Australia Small (WAS), Spermonde Large (SpL), Spermonde Small (SpS)) and previously described *Amphisorus* species.

Morphotype	WAL	WAS	SpL	SpS	*A*. *kudakajimaensis* (Gudmundsson, 1994)	*A*. *sauronensis* Lee et al. 2007	*A*. *hemprichi* Ehrenberg, 1839
	28 specimens	9 specimens	32 specimens	19 specimens			
Proloculus (μm)	290–464 (372)	195–255 (225)	264–533 (378)	144–250 (178)	~380	~330	150–340 ***)
**Alfa**	159.7–175.0 (167.3; 33)	63.0–77.2 (70.0; 9)	211.2–233.8 (227; 26)	130.0–139.3 (134.9; 19)	~200 **)	~175–180 *)	125–140
**Apertures**	rimmed	smooth	rimmed	smooth	rimmed	smooth	rimmed
**Marginal apertures**	elongated, usually 2–5 times as long as wide, parallel.	Elongated in two alternating rows	elongated, usually not more than 3 times as long as wide, oblique.	Round, in two alternating rows	elongated, ellipsoidal to y shaped.	'vary in shape and size from small and nearly round (~30 μm) to long and sinuous.	two alternating rows of round, ellipsoidal to occasionally y-shaped
**Median apertures**	in depressions, initially round or slightly elongated, getting longer and occasionally irregulary y or x shaped.	occasionally with a single round median aperture (1 in 5–10 marginal apertures)	At most 3–5 rows of median apertures; elongated, kidney shaped and occasionally y shaped	rarely with a single median aperture	3–5 lines of irregularly placed median apertures elongated, ellipsoidal to y or X shaped.	'exhibiting a wide range of from irregular to perfectly round and ~30 μm in diameter'; 1–3 rows of median apertures *)	not mentioned
**Axial section**	median skeleton regularly increasing in thickness; multiple connections to previous chamber per chamberlet; chamberlets frequently are discontinuous from one side to the other side of the test	Dominated b alternating walls of marginal apertures, with a rare irregularity in the center	median skeleton regularly increasing in thickness; 3–5 connections to previous chamber per chamberlet; most if not all chamberlets are continuous from one side to the other side of the test	duplex immediately following the embryon	median skeleton regularly increasing in thickness; 3–5 connections to previous chamber per chamberlet; most if not all chamberlets are continuous from one side to the other side of the test	median skeleton increasing in thickness, in the marginal part ~1/3 of the thickness of the test. Median skeleton usually with 2, sometime 1 or 3 connections to adjoining chamberlets	duplex develops in 11-17th annular chamber
**Lateral chamberlets**	higher than wide, honeycomb shaped	as wide as high; flabelliform	higher than wide, honeycomb shaped	as wide as high; flabelliform	paralell side, as high as wide to 1.5* as high as wide	flabelliform	as wide as high; flabelliform

*) based on topotypic material from Lizard Island ([45]; **) from figured specimens in type description; ***) [46] ***)).

WAL morphotype specimens were observed in two sites in the Shark Bay area (‘Bush Bay’, 2 specimens; ‘Whale Road Beach’, 8 specimens), and in ‘Jurien Bay’ (one specimen). Morphologically identical dry specimens for comparison were available from Little Armstrong Bay (Rottnest Island, WA, Australia). We observed WAS morphotype specimens in all sites in Western Australia, except at the site ‘Whale Road Beach’. Our SpL morphotype specimens were from the barrier reef in the Spermonde Archipelago, but morphologically identical specimens occur in the outer zone of the midshelf reefs as well [44], whereas the SpS specimens in this study were from Pulau Karanrang, a nearshore reef in the Spermonde Archipelago. Morphologically identical specimens occur in the entire inner part of the midshelf zone [44]. We compare the four morphotypes identified in this study to published descriptions of *A*. *kudakajimaensis*, *A*. *sauronensis*, and *A*. *hemprichii* in Table 1.

### Molecular identification of *Amphisorus* and dinoflagellate symbionts

Sequencing of analysis of the 38 *Amphisorus* specimens (1 SpS morphotype, 3 SpL morphotype, 11 WAL morphotype, 23 WAS morphotype) resulted in 910,002,258 raw reads. The negative control contained 110,532 reads (corresponding to 0.01% of all reads). Illumina platforms are known to produce tag switching [47, 48], and a small proportion of reads is commonly found in negative controls. As the number of reads observed in our negative control was low, we did not suspect contamination. After strict quality filtering with removal of all bases with phred score of <15 and reads shorter than 100bp length, 495,195,764 reads were retained (all read counts per sample: S2 Table in S3 File). A 4,307 basepair fragment spanning the partial 18S rRNA, 5.8S rRNA, and partial 28S rRNA was recovered for the 38 molecularly analysed *Amphisorus* specimens. The minimum coverage was 33.9 (sample WRB7), and the maximum coverage 1611.5 (sample WRB12). The average coverage across all samples was 320.1 (coverage per sample: S3 Table in S3 File). Alignment of specimens from this study to the available 526bp *Amphisorus hemprichii* 18S rRNA reference sequences from Rottnest Island showed that all samples collected along the coast of Australia were 100% identical to these references. All specimens from Indonesia showed a 99.048% similarity to the references form Rottnest Island. Alignment of the V4 region of the 18S rRNA of our Australian *Amphisorus* sequences to the available sequences from outside of Western Australia showed that the maximum genetic difference ranged from 0.5% (*Amphisorus hemprichii*, accession number AJ843163.1, Lizard Island, Australia) to 9% (unidentified *Amphisorus* AJ404313.1 from Eilat, Israel). All but the latter specimen had a maximum genetic difference of 2.2% to our specimens from Western Australia. Our specimens from Indonesia showed differences between 1.8% (*Amphisorus kudakajimaensis* AJ843138.1, Guam) to 10% (unidentified *Amphisorus* AJ404313.1 from Eilat, Israel) to existing references.

Three clades of dinoflagellate symbionts were identified. *Fugacium* Fr5 [14], was found in all specimens except two from Indonesia (IL2, morphotype SpS; IL7, morphotype SpL). The recovered 28S rRNA fragment had a length of 606bp, and the mean coverage was 185.2 (coverage per sample: S4 Table in S3 File). Another clade of dinoflagellate, Fr3 [14], was found in the four specimens from Indonesia with a mean coverage of 79. *Cladocopium* (Clade C) [14] was found in the four *Amphisorus* specimens from Indonesia, but the mean coverage was low at 7.7. Only *Fugacium* Fr5 was further analysed due to its presence in a high number of *Amphisorus* specimens.

### Accordance of *Amphisorus* morphotype and genotype

**Fig 4:**
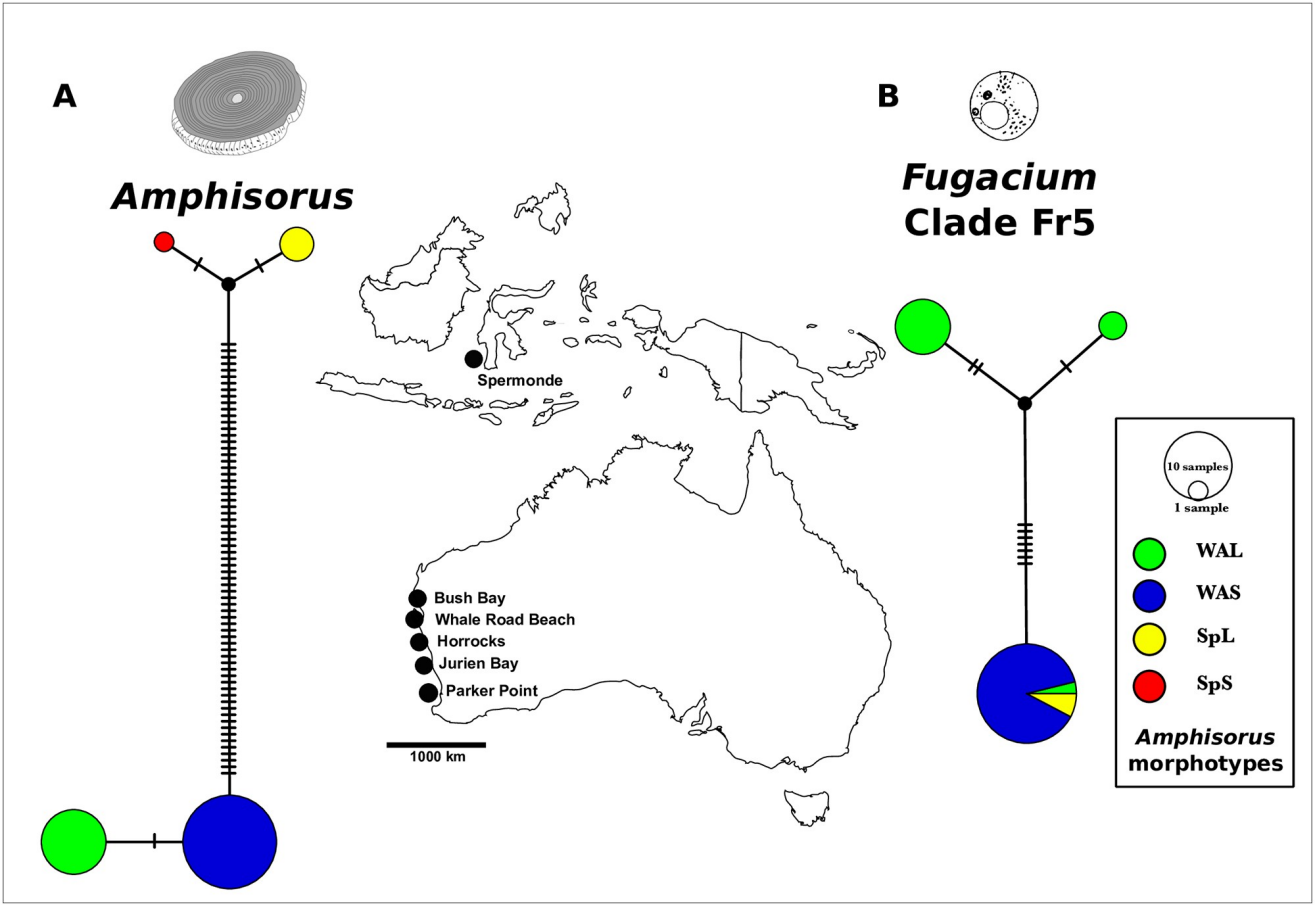
Map showing the sampling locations in Australia and Indonesia, and median-joining networks showing the four *Amphisorus* genotypes (based on a 4,307bp fragment spanning the partial 18S rRNA, 5.8S rRNA, and partial 28S rRNA), and three *Fugacium* Fr5 genotypes (based on a 606bp 28S rRNA fragment). Each circle in the network represents a distinct genotype. Colours indicate the morphotype of analysed *Amphisorus* specimens. Each black bar indicates one substitution. Circle size corresponds to the number of specimens.

Molecular analysis of the 18S- 5.8S-28S rRNA fragment of 38 *Amphisorus* specimens (1 SpS morphotype, 3 SpL morphotype, 11 WAL morphotype, 23 WAS morphotype) demonstrated that the four identified *Amphisorus* morphotypes correspond to four genotypes. Morpho-/genotype Spermonde Large (SpL) differed from morpho-/genotype Spermonde Small (SpS) by two substitutions. All 23 specimens of morphotype Western Australia Small (WAS) shared the same genotype. A second genotype, with one substitution difference, comprised all 11 specimens of the Western Australia Large (WAL) morphotype. The WAS genotype and the SpS and SpL genotypes, respectively, differed by 35 substitutions (Fig 4A). All morphotypes had a nucleotide diversity π of 0.

The analysed *Amphisorus* specimens harboured three genotypes of the dinoflagellate symbiont *Fugacium* Fr5. The most common genotype was found in all 23 *Amphisorus* specimens of the WAS morphotype, in two SpL morphotype specimens (IL6, IL9), and in one WAL specimen (JB8). This genotype was eight substitutions different from a genotype found in two WAL specimens (WRB9, WRBB11), and 9 substitutions different to a genotype found in the remaining nine WAL morphotype specimens. The latter two genotypes differed by three substitutions (Fig 4B). Nucleotide diversity π of *Fugacium* Fr 5 was calculated per *Amphisorus* morphotype, and was 0 for morphotype WAS, 0.001 (4 parsimony-informative sites) for morphotype WAB, and 0 for morphotype SpL.

### Microbial community analysis

**Fig 5:**
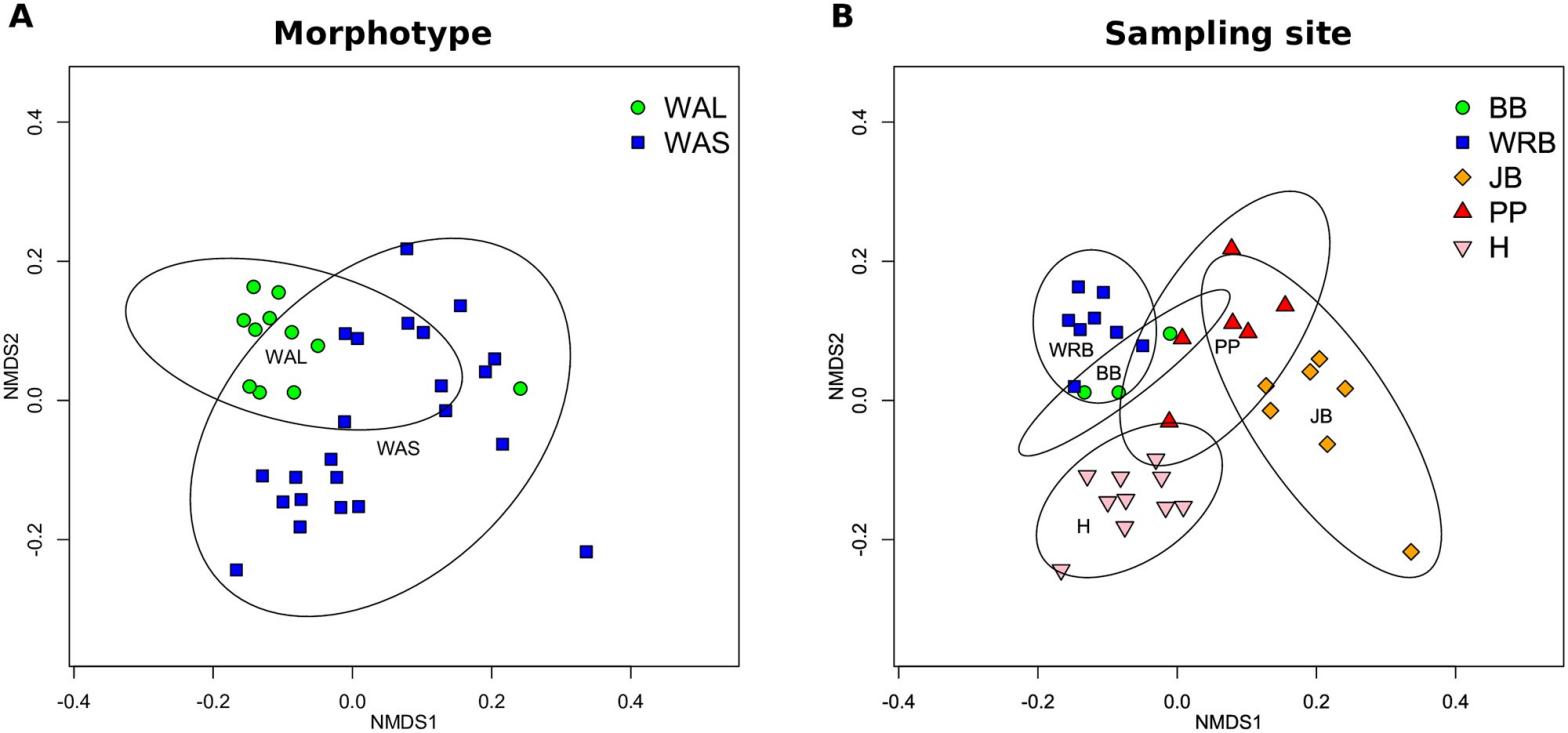
NMDS plots showing the community composition of 26 *Amphisorus*-associated bacteria based on Bray-Curtis distance. A) Differences in bacterial community composition between WAS and WAL morphotype. B) Differences in bacterial community composition between sampling sites.

The analysis on microbial communities focused on the 11 WAL and 23 WAS morphotype specimens from Western Australia. The megahit assembly resulted in 713,321 contigs, of which 324,315 were identified as prokaryotic by the EukRep algorithm. Binning and anvi’io analysis revealed 26 metagenome assembled genomes (MAGs) with a completeness of >50% and a redundancy of <5%. Of these MAGs, 22 had a completeness of >90%, corresponding to high-quality draft genomes [36]. In total 10 different taxonomic orders were identified. Out of the 26 MAGs, 24 could be assigned to a taxonomic order. The Granulosicoccales were the most common order with five MAGs, followed by Flavobacteriales and Rhizobiales with four MAGs each (see S5 Table in S3 File for table of taxonomic annotation, abundance, MAG completeness and gene redundancy). Adonis analysis showed that *Amphisorus* morphotype explained overall bacterial community composition with R^2^ = 0.19 (p = 0.001). The NMDS plot (stress: 0.12) showed that WAL morphotype specimens form a distinct cluster, with the exception of the single WAL specimen (JB8) found at the Jurien Bay sampling site (Fig 5A). Sampling site was a stronger predictor of community composition with R^2^ = 0.63 (p = 0.001). The NMDS plot showed that *Amphisorus* specimens from the same sampling site clustered closer together, i.e. had more similar bacterial communities, although some overlap between sampling sites occurs (Fig 5B).

## Discussion

In this study, we combined micro CT imaging and shotgun metagenomics to assess the morphological and molecular diversity of the large benthic foraminifera *Amphisorus* along the coast of Western Australia. We compared specimens from Western Australia with specimens from Indonesia, and enhanced the integrative approach by studying the genetic diversity of dinoflagellate symbionts, and by assessing the community composition of *Amphisorus-* associated bacteria. Gaining deeper and precise insights into the taxonomic and genetic diversity of foraminifera is crucial for assessing their true biodiversity and ecology, knowledge that can be used in conservation planning and applications like biomonitoring [49], which is increasingly important in times of global biodiversity loss. We identified four distinct *Amphisorus* morphotypes, which correspond to four genotypes. Further we showed that the genotypes of the common dinoflagellate symbiont *Fugacium* Fr5 largely correspond to host morpho- and genotypes. The *Amphisorus*-associated bacterial community, even though different between *Amphisorus* morphotypes, seems to be mainly shaped by the environment.

### Diversity of *Amphisorus*

Until the discovery of *A*. *kudakajimaensis* [19], originally described as *Marginopora*, and *A*. *sauronensis* [20], the genus *Amphisorus* has been regarded as monotypic, consisting of a single species with a circumtropical distribution [19, 50]. To date, large, complex *Amphisorus*- like specimens from Southeast Asia and the Pacific are thought to belong to *A*. *kudakajimaensis* [50, 51]. whereas those around Australia are referred to as *A*. *sauronensis* (e.g., [19]). However, there is large variability within populations assigned to *A*. *kudakajimaensis*, probably including several yet unrecognised species [52]. In both sampling areas (Western Australia and Indonesia), we encountered two co-occurring morphotypes. Previously, Parker also found two morphotypes of *Amphisorus* in Western Australia [53], but he could not rule out that they were two morphs of the same taxon given the range of intermediate specimens he observed.

Using shotgun metagenomic sequencing and analysis of a 4,307 base pair fragment spanning the 18S- 5.8S- and 28S rRNAs, we identified four genotypes of *Amphisorus*, which correspond to the four morphotypes. Genetic differences between morpho-/ genotypes from Indonesia (SpS and SpL) and those from West Australia (WAS, WAL) were comparatively large with 35 substitutions. On the contrary, genetic differences between SpS and SpL and WAS and WAL, respectively, were small with three respectively one substitution. The small number of analysed specimens and the small genetic differences between morphotypes from the same region does not allow to reliably define new foraminifera species based on molecular data (e.g., [5]), and we point out that minimal genetic differences as found in this study can easily be lost by single mutation events.

Foraminifera reproduce sexually and asexually, with the different life-cycles resulting in different morphologies within species [54, 55]. The asexual generation has a large proloculus (macrosphere or A-form) and in most species a smaller test diameter, whereas the sexual generation (microsphere or B-form) has a small (usually <50–60 μm) proloculus and in most species a larger test diameter. The complicating factor is that in some species, including in the closely related genera *Marginopora* and *Sorites* [56, 57], the sexual reproduction is suppressed or very rare, resulting in an alternation of asexual generations (A1-A2 which are both macrospheric), in which there can be small morphological differences between the A1 and A2 generation, usually reflected in the diameter of the proloculus and deuteroconch [58]. Therefore, our findings beg the question whether the WAS and WAL and SpS and SpL macrospheric morphotypes can be interpreted as different generations of a single Australian respectively Indonesian species. We conclude that this is unlikely for a number of reasons.

Firstly, our genetic data support the morphological findings and underpin that different morphotypes do not represent different generations or are the result of phenotypic plasticity, which has been found in foraminifera earlier [59, 60]. Secondly, there is a consistent morphological difference not related to the reproduction mode. SpS and WAS both have unrimmed apertures, whereas their larger companions SpL and WAL have rimmed apertures. Thirdly but more indirectly, the WAL and SPL embryon diameter is twice that of WAS and SpS respectively. In comparison, in the closely related *Sorites orbiculus* no difference in embryon diameter was observed [56]. Direct observations of morphological differences between A1 and A2 are rare, and generations may be inferred from embryon diameter distribution in life populations or the sediment [61]. In our material, there appears to be a bimodal distribution in α (see Fig 2A) in SpL, allowing the possibility that SpL includes both A1 and A2 generations. Thus, we conclude that we have identified four distinct morphotypes, two of which co-occur in Australia, and two in Indonesia.

Having established the presence of four morphotypes in our collections, we tried to match these with existing named species. WAL is most similar to *A*. *sauronensis*, but differs by the rimmed apertures and the less complex shape of the median apertures. SpL is most similar to *A*. *kudakajimaensis*, but differs in the shape of the lateral chamberlets (narrow vs. wide). Both WAS and SpS have the simple morphology lacking the intermediate skeleton and median apertures, which makes them most similar to *A*. *hemprichii*. The comparison with published descriptions of *Amphisorus kudakajimaensis* [19], *A*. *sauronensis* [20], and *A*. *hemprichi* [21] showed that the morphotypes identified by us differ from the described species.

We point out that even though the comparison with reference 18S data shows that our *Amphisorus* specimens are highly similar to the existing reference sequence of *A*. *hemprichii* from Western Australia, it is possible that ‘basetypes’ as defined by [5]. i.e. several genetic variants of the 18S rRNA or other genes in one specimen, are also present in *Amphisorus*. The shotgun metagenomic approach and assembly of short reads with up to 5% mismatch allowed accounts for this, but merges potential basetypes into a consensus that would be similar to a ‘basegroup’ defined by [5]. The 18S rRNA is commonly and successfully used to study genetic diversity and taxonomy of various groups of foraminifera [3, 62–65]. However, it has been shown that in some species the 18S fragment can be too conserved to distinguish closely related species [66]. In congruence with that, the small, but consistent genetic difference we found between the WAS and WAL *Amphisorus* morphotypes from Western Australia was not found in the 18S rRNA, but in the ITS 2 region between the 5.8S and 28S rRNA. In the future, longer fragments, but also other genes should be routinely utilised for studies on genetic diversity of *Amphisorus* and other closely related Foraminifera, a task that might be achieved by utilising shotgun metagenomics. However, public reference databases contain only a few foraminiferal genes, which makes it difficult to assess how informative other genes are for the identification of closely related Foraminifera species. Our efforts to recover the α—tubulin and β- tubulin genes, which were used in previous studies on foraminifera [67], failed, meaning either that available references are too different from genes present in our specimens, or that sequencing depth did not allow to recover genes present in low copy numbers. Foraminifera genomes are known to contain highly repetitive elements and introns [68, 69], which can make discovery of genes challenging. Gene prediction and identification in protists is often difficult, also due to the lack of reference genomes [70]. Mining of genes from available Foraminifera transcriptomes [68, 71]) proved unsuccessful, potentially due to the presence of introns and the short read length used in our study. We therefore suggest the targeted sequencing of full foraminiferal genomes using long-read sequencing platforms like PacBio or Nanopore, and targeted sequencing of transcriptomes to build up comprehensive molecular reference libraries spanning a wide range of species and genes and improve understanding of Foraminifera genomics. However, the small size of many Foraminifera species makes obtaining high-quality DNA and RNA for genome and transcriptome sequencing from single individuals challenging. These issues could be overcome by single-cell genome and transcriptome sequencing protocols for protists [72, 73]. although the presence of symbionts and the calcified test of Foraminifera might pose a challenge for efficient DNA/RNA extraction, assembly and annotation. Further studies based on a higher number of genetic markers could help determine the species status of the four identified *Amphisorus* types by allowing to study a large number of potentially variable and informative genes.

### Diversity of the dinoflagellate symbiont *Fugacium* Fr5

We enhanced the studies on *Amphisorus* diversity by studying whether occurrences and genotypes of the dinoflagellate symbionts match the described *Amphisorus* morpho-/ genotypes. We found three different dinoflagellate symbionts in the studied *Amphisorus* specimens. *Fugacium* Fr5 was the most abundant, and present in the *Amphisorus* morphotypes WAS, WAL and SpL. *Fugacium* Fr5 is a known symbiont of *Amphisorus* from Eilat (Israel) and the Maldives [74]. The second dinoflagellate, clade Fr3, was found only in the specimens from Indonesia. This clade has previously been found in *Amphisorus* from Guam [74] and in sediment and water from the Great Barrier Reef [75]. The least abundant dinoflagellate in our dataset, *Cladocopium* (Clade C), was also found only in specimens from Indonesia. This clade is a widely distributed dinoflagellate symbiont known from multiple marine taxa [14]. Our results therefore show that Australian and Indonesian *Amphisorus* morpho- /genotypes differ in the number of symbiont taxa they harbour, further underlining the difference between these *Amphisorus* morpho- and genotypes. Dinoflagellate symbionts have been found to be host-specific to some degree [51, 76, 77]. In line with that, our results showed that the three identified *Fugacium* Fr5 [14] genotypes correspond to a large extent with the host *Amphisorus* morpho-/ genotypes. However, a strict host specificity was not found, and we point out that previous studies showed that the dominant dinoflagellate symbiont type in a population can change over time [76]. Further studies on a larger number of specimens are needed to understand ecology and genomics of dinoflagellate symbionts and their hosts, and reference data comprising dinoflagellate strains and information on the host species and genotype should be generated by future studies.

### Community composition of *Amphisorus*- associated bacteria

Previous studies have discussed the ecological and evolutionary importance of the microbiome in LBF [1, 78]. Our data on the *Amphisorus* morphotypes WAL and WAS from Western Australia shows that community composition of *Amphisorus*- associated bacteria significantly differs between host morpho/genotypes. However, sampling location was a stronger predictor of microbial community composition, meaning that *Amphisorus* specimens from the same sampling site harbour more similar microbial communities. Therefore, we infer that local habitat (as opposed to the host organism) is a stronger driver of bacterial community composition found in association with *Amphisorus*, as also observed in other LBF species [17, 18]. This might be due to the fact that LBF feed on microbial organisms [79], whose community composition can be strongly linked to microhabitat [80]. Further, the identified bacterial taxonomic groups have previously been found in seawater samples and marine sediments [81–84], rendering it more likely that the identified bacteria were ingested as food organisms or formed a biofilm on the surface of the foraminifera. However, due to the lack of reference sequences of bacteria associated with *Amphisorus*, we cannot exclude that at least some of the identified bacteria are symbionts. Furthermore, it is possible that symbiotic bacterial taxa were present in our samples, but were not successfully assembled to MAG level due to limited sequencing depth or high fragmentation of the genomes. To conclusively answer whether different *Amphisorus* morpho-/ genotypes harbour distinct bacterial communities, future studies comprising a higher number of analysed specimens and different species should build up a reference library of foraminifera- associated bacterial taxa. Microbial taxa found in the foraminifera should be compared with those found in the sediment and seawater to disentangle potentially symbiotic taxa and organisms that live in foraminifera’s environment and are potential food organisms.

## Conclusion

Traditional species concepts in foraminiferal have partitioned morphological types into distinct species based on test shape (morphospecies). With the advance of genetic studies initially more and more evidence was presented that demonstrates that individual morphospecies can be actually complexes of several discrete genetic types (genotypes), including distinct cryptic species [85]. However, detailed biometric analysis revealed, for example in *Orbulina*, that these cryptic species had consistent morphological differences [86]. The overall challenge lies in separating environmentally induced intraspecific variation from interspecific morphologic variation. Likewise there is a large difference in the amount of genetic variability between sites, populations and within species in benthic foraminifera. For example, the 18S SSU rDNA fragment in *Amphistegina lobifera* is hypervariable within sites, and populations, and showed six hierarchically organised genotypes [87]. This is in strong contrast to our results in *Amphisorus* in which we find no intrapopulation and site, as well as very limited interspecific variation despite strong morphological evidence of species level differentiation. This highlights the importance of integrating state-of-the-art morphological analyses and molecular techniques targeting the foraminiferal host, its dinoflagellate symbionts and associated bacteria to fully understand the diversity of large benthic foraminifera. We argue that an integrative approach as applied in this study can help delineate yet undescribed taxa, and is especially promising for the study of taxa for which morphological and molecular identification is challenging due to a limited morphological and genetic references. By using this approach we discover further evidence that the species richness in the genus *Amphisorus* is underexplored, and that detailed studies over its entire range are needed to fully understand its diversity.

## Supporting information

S1 Fig *Amphisorus* in situ A) morphotype Spermonde Large (SpL) occurs predominantly on coral rubble and calcareous algae. B) morphotype West Australia Large (WAL) is found predominantly on seagrass leaves.
(PDF)

S2 Fig Virtual horizontal cross sections through the A forms of the four morphotypes of *Amphisorus* recognised in this paper. A) Spermonde Small (SpS) (specimen PKKW3_A6). B) Spermonde Large (SpL) (specimen UPG93RF3_8). C) West Australia large (WAS). (specimen Rottnest_A3). D) West Australia Large (WAL). (specimen Wooramel_A1). Scale bar = 200 μm.
(PDF)

S1 File Commands used for assembly and refining of prokaryote MAGs from *Amphisorus*. Commands shown for one sample.
(PDF)

S2 File R script used for analysis of MAG abundance. (PDF)

S3 File (PDF)
