# Supplementary material for: Integrating morphology and metagenomics to understand taxonomic variability of Amphisorus (Foraminifera, Miliolida) from Western Australia and Indonesia
Source: PLoS One. 2021 Jan 4;16(1):e0244616. doi: 10.1371/journal.pone.0244616 (PMC7781389; doi:10.1371/journal.pone.0244616)
Supplement: S1 Fig — Amphisorus in situ A) morphotype Spermonde Large (SpL) occurs predominantly on coral rubble and calcareous algae. B) morphotype West Australia Large (WAL) is found predominantly on seagrass leaves. (PDF) [file pone.0244616.s001.pdf]

Integrating morphology and metagenomics to understand taxonomic variability of *Amphisorus* (Foraminifera, Miliolida) from Western Australia and Indonesia

Authors: Jan-Niklas Macher, Martina Prazeres, Sarah Taudien, Jamaluddin Jompa, Aleksey Sadekov, Willem Renema

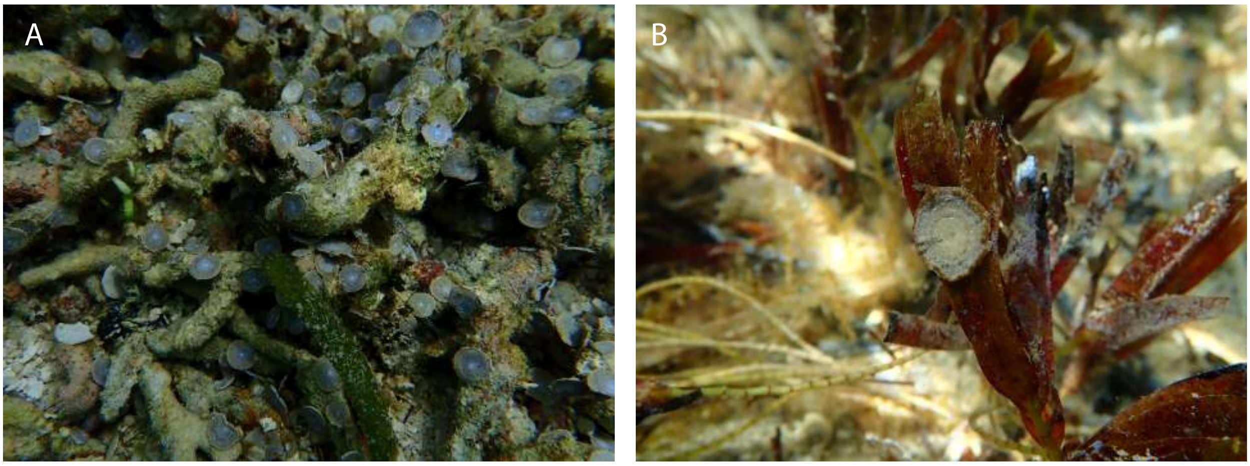

Supplementary figure S1: *Amphisorus* in situ A) morphotype Spermonde Large (SpL) occurs predominantly on coral rubble and calcareous algae. B) morphotype West Australia Large (WAL) is found predominantly on seagrass leaves.
