# Supplementary material for: Integrating morphology and metagenomics to understand taxonomic variability of Amphisorus (Foraminifera, Miliolida) from Western Australia and Indonesia
Source: PLoS One. 2021 Jan 4;16(1):e0244616. doi: 10.1371/journal.pone.0244616 (PMC7781389; doi:10.1371/journal.pone.0244616)
Supplement: S2 Fig — A) Spermonde Small (SpS) (specimen PKKW3_A6). B) Spermonde Large (SpL) (specimen UPG93RF3_8). C) West Australia large (WAS). (specimen Rottnest_A3). D) West Australia Large (WAL). (specimen Wooramel_A1). Scale bar = 200 μm. (PDF) [file pone.0244616.s002.pdf]

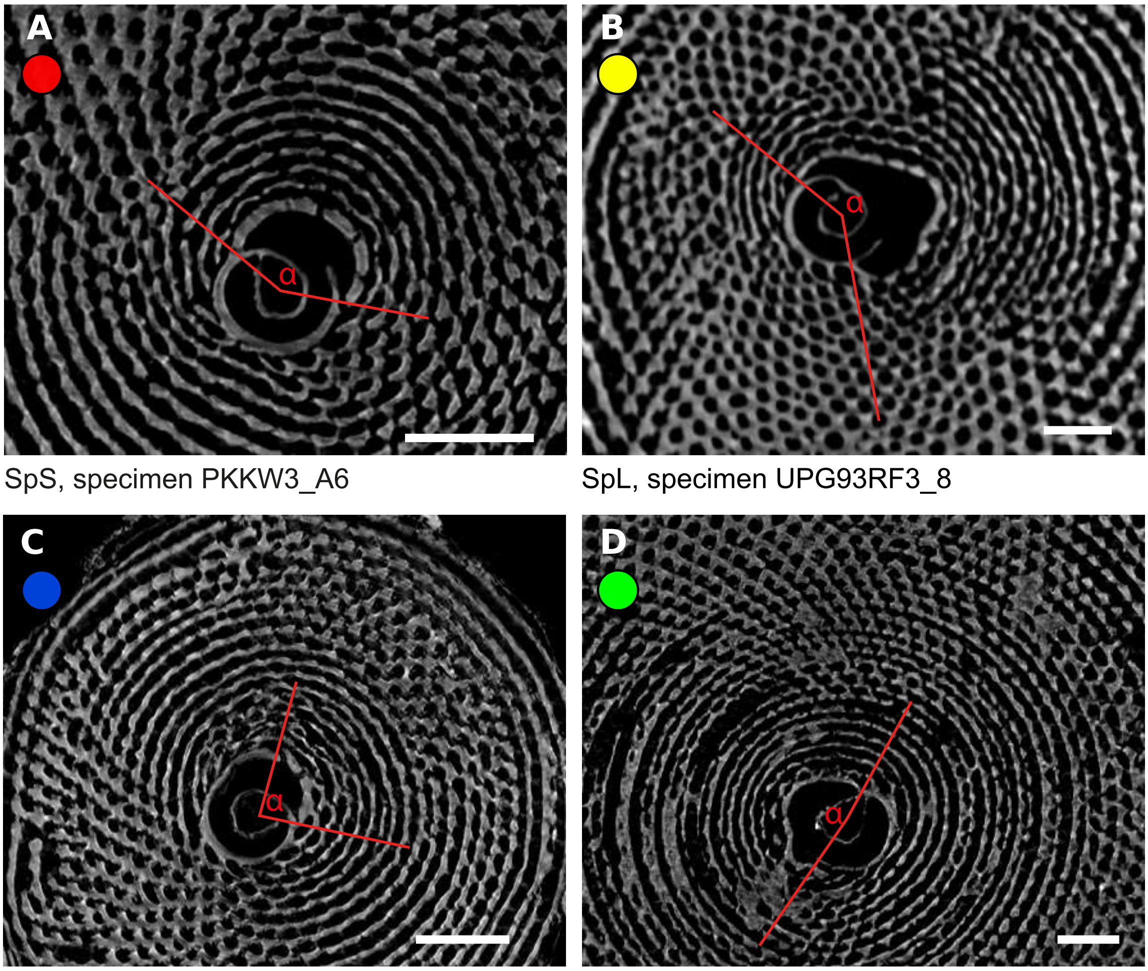

SpS, specimen PKKW3_A6

SpL, specimen UPG93RF3_8

WAS, specimen Rottnest_A3

WAL, specimen Wooramel_A1

Supplementary figure S2: Virtual horizontal cross sections through the A forms of the four morphotypes of *Amphisorus* recognised in this paper. A) Spermonde Small (SpS) (specimen PKKW3_A6). B) Spermonde Large (SpL) (specimen UPG93RF3_8). C) West Australia large (WAS). (specimen Rottnest_A3). D) West Australia Large (WAL). (specimen Wooramel_A1). Scale bar = 200 µm
