# Supplementary material for: Integrating morphology and metagenomics to understand taxonomic variability of Amphisorus (Foraminifera, Miliolida) from Western Australia and Indonesia
Source: PLoS One. 2021 Jan 4;16(1):e0244616. doi: 10.1371/journal.pone.0244616 (PMC7781389; doi:10.1371/journal.pone.0244616)
Supplement: S1 File — Commands shown for one sample. (PDF) [file pone.0244616.s003.pdf]

Integrating morphology and metagenomics to understand taxonomic variability of *Amphisorus* (Foraminifera, Miliolida) from Western Australia and Indonesia

Authors: Jan-Niklas Macher, Martina Prazeres, Sarah Taudien, Jamaluddin Jompa, Aleksey Sadekov, Willem Renema

S1 File: Commands used for assembly and refining of prokaryote MAGs from *Amphisorus*. Commands shown for one sample.

##MEGAHIT Assembly
megahit -1 Sample1_1_trimmed.fastq.gz -2 Sample1_2_trimmed.fastq.gz --min-contig-len 2000 -o Amphisorus-RAW.fa --num-cpu-threads 16 --presets meta-large

##Identify Eukaryota and Prokaryota
EukRep -i Amphisorus-RAW.fa -o Amphi_Eukaryota.fa --prokarya Amphi_Prokaryota.fa --min 2000

##Reformat names
anvi-script-reformat-fasta Amphi_Prokaryota.fa -o Amphi_Prokaryota_fixed_min2000.fa –l 2000 --simplify-names

##Make new folder for mapping
mkdir 04_MAPPING

##build an index for the contigs
bowtie2-build Amphi_Prokaryota_fixed_min2000.fa 04_MAPPING/contigs --threads 32

##Mapping for bam and sorting
bowtie2 --threads 32 -x 04_MAPPING/contigs -1 Sample1_1_trimmed.fastq.gz -2 Sample1_2_trimmed.fastq.gz -S 04_MAPPING/Sample1.sam
samtools view -F 4 -bS 04_MAPPING/ Sample1.sam > 04_MAPPING/ Sample1-RAW.bam
anvi-init-bam 04_MAPPING/ Sample1-RAW.bam -o 04_MAPPING/ Sample1.bam
rm 04_MAPPING/ Sample1.sam 04_MAPPING/ Sample1-RAW.bam

##Create the contigs database
anvi-gen-contigs-database -f Amphi_Prokaryota_fixed_min2000.fa -o Amphi_Prokaryota_fixed_min2000.db -L 20000

##run hmms to identify genes
anvi-run-hmms -c Amphi_Prokaryota_fixed_min2000.db--num-threads 32

##Display contigs stats (number of genes etc.)
anvi-display-contigs-stats Amphi_Prokaryota_fixed_min2000.db

##open new chrome tab and go to
http://localhost:8080

##Profiling BAM files
anvi-profile -i 04_MAPPING/Sample1.bam -c Amphi_Prokaryota_fixed_min2000.db----min-contig-length 2000 --num-threads 32 -o 05_Profiles/Sample1 -W --sample-name Sample1

##Merge profiles
anvi-merge 05_Profiles/Sample1/PROFILE.db 05_Profiles/Sample2/PROFILE.db -o Amphi-SAMPLES-MERGED -c Amphi_Prokaryota_fixed_min2000.db

##Binning
anvi-cluster-contigs -c Amphi_Prokaryota_fixed_min2000.db -p Amphi-SAMPLES-MERGED/PROFILE.db --clusters 100 -C CONCOCT -T 40 --driver CONCOCT --just-do-it
anvi-cluster-contigs -c Amphi_Prokaryota_fixed_min2000.db -p Amphi-SAMPLES-MERGED/PROFILE.db -C METABAT2 -T 32 --driver metabat2 --just-do-it
anvi-cluster-contigs -c Amphi_Prokaryota_fixed_min2000.db -p Amphi-SAMPLES-MERGED/PROFILE.db -C MAXBIN2 -T 40 --driver maxbin2 --just-do-it
anvi-cluster-contigs -c Amphi_Prokaryota_fixed_min2000.db -p Amphi-SAMPLES-MERGED/PROFILE.db -C DASTOOL --source-collections CONCOCT,METABAT2,MAXBIN2 -T 32 --driver dastool --just-do-it --search-engine diamond

##Get a summary
anvi-summarize -c Amphi_Prokaryota_fixed_min2000.db -p Amphi-SAMPLES-MERGED/PROFILE.db -C CONCOCT -o SET-SUMMARY-CONCOCT
anvi-summarize -c Amphi_Prokaryota_fixed_min2000.db -p Amphi-SAMPLES-MERGED/PROFILE.db -C METABAT2 -o SET-SUMMARY-METABAT2
anvi-summarize -c Amphi_Prokaryota_fixed_min2000.db -p Amphi-SAMPLES-MERGED/PROFILE.db -C MAXBIN2 -o SET-SUMMARY-MAXBIN2
anvi-summarize -c Amphi_Prokaryota_fixed_min2000.db -p Amphi-SAMPLES-MERGED/PROFILE.db -C DASTOOL -o SET-SUMMARY-DASTOOL

##Visualise
anvi-interactive -p Amphi-SAMPLES-MERGED/PROFILE.db -c Amphi_Prokaryota_fixed_min2000.db -C CONCOCT
anvi-interactive -p Amphi-SAMPLES-MERGED/PROFILE.db -c Amphi_Prokaryota_fixed_min2000.db -C METABAT2
anvi-interactive -p Amphi-SAMPLES-MERGED/PROFILE.db -c Amphi_Prokaryota_fixed_min2000.db -C MAXBIN2
anvi-interactive -p Amphi-SAMPLES-MERGED/PROFILE.db -c Amphi_Prokaryota_fixed_min2000.db -C DASTOOL

##open new chrome tab and go to
http://localhost:8080

##Refine BINs if needed (example)

```
anvi-refine -c Amphi-SAMPLES-MERGED/PROFILE.db \
        -p Amphi-SAMPLES-MERGED/PROFILE.db \
        -C CONCOCT \
        -b Bin_41
```

##open new chrome tab and go to

http://localhost:8080

##Rename bins, remove low quality bins, and store in FINAL folder

```
anvi-rename-bins -c Amphi_Prokaryota_fixed_min2000.db \
              -p Amphi-SAMPLES-MERGED/PROFILE.db \
              --collection-to-read DASTOOL \
              --collection-to-write DASTOOL_FINAL \
              --call-MAGs \
              --size-for-MAG 2 \
              --min-completion-for-MAG 0 \
              --max-redundancy-for-MAG 0 \
              --prefix WRB \
              --report-file DAS_renaming_bins.txt
```

##Binning summary for each metagenomic set.

```
anvi-summarize -c Amphi_Prokaryota_fixed_min2000.db \
             -p Amphi-SAMPLES-MERGED/PROFILE.db \
             -C DASTOOL_FINAL \
             -o Amphi-SUMMARY-FINAL_DAS
```

##Visualise

```
anvi-interactive -p Amphi-SAMPLES-MERGED/PROFILE.db -c
Amphi_Prokaryota_fixed_min2000.db -C DASTOOL_FINAL
```

##open new chrome tab and go to

http://localhost:8080

###Get all fasta files and store in Fastas folder

```
cd Amphi-SUMMARY-FINAL_DAS/bin_by_bin
mkdir Fastas
mv **/*.fa Fastas
cd ../..
```
