# Supplementary material for: Integrating morphology and metagenomics to understand taxonomic variability of Amphisorus (Foraminifera, Miliolida) from Western Australia and Indonesia
Source: PLoS One. 2021 Jan 4;16(1):e0244616. doi: 10.1371/journal.pone.0244616 (PMC7781389; doi:10.1371/journal.pone.0244616)
Supplement: S2 File — (PDF) [file pone.0244616.s004.pdf]

S2 File: R script used for analysis of MAG abundance

```
library(picante)

library(ggpubr)


setwd("8_Anvio_Prokaryota")

comm <-
read.csv("Amphi_MAG_Abundance_relative_per_sample.csv",header=TRUE,
row.names=1,sep=";")

head(comm)
comm<-t(comm)
head(comm)

#Test skewness
library(moments)

hist(comm,col='red')
qqnorm(comm)
skewness(comm)

#Apply log transformation
comm <- decostand(comm, "log")

######

metadata <- read.csv("Sites_final_WAB_WAS.csv", header = TRUE,
 row.names = 1, sep=";")
# take a peek at the data
head(metadata)

ls()
all.equal(rownames(comm), rownames(metadata))

#Boxplot
boxplot(specnumber(comm) ~ metadata$SamplingSite, ylab = "# of
 species")
# statistical test of difference
t.test(specnumber(comm) ~ metadata$SamplingSite)

# calculate Bray-Curtis distance
comm.bc.dist <- vegdist(comm, method = "bray", binary = F)
# cluster communities using average-linkage algorithm
comm.bc.clust <- hclust(comm.bc.dist, method = "average")
# plot cluster diagram
plot(comm.bc.clust, ylab = "Bray-Curtis dissimilarity")
```

```
# Adonis
adonis(comm.bc.dist ~ SamplingSite, data = metadata)
adonis(comm.bc.dist ~ Morphotype, data = metadata)

############

#Ordination
#metaMDS
comm.bc.mds <- metaMDS(comm, dist = "bray", binary =F,  k = 2,
 trymax = 1000, autotransform = T)

#Stress plot
stressplot(comm.bc.mds)

# plot site scores as text
ordiplot(comm.bc.mds, display = "sites", type = "text")


#plot
mds.fig <- ordiplot(comm.bc.mds, type = "none", xlim=c(-1.2,1.2),
 ylim=c(-1.2,1.2))

# plot the samples, color by morphotype
points(mds.fig, "sites", pch = 21, cex=1.5, col = "black",
 bg="green", select = metadata$Morphotype ==
         "WAL")
points(mds.fig, "sites", pch = 22, cex= 1.5, col = "black",
 bg="blue", select = metadata$Morphotype ==
         "WAS")
 legend("topleft"
  , inset = c(0.81,0.01),
  , cex = 1.5,
  , bty = "n",
  , legend = c("WAL", "WAS"),
  , text.col = c("black"),
  , col = c("black"),
  , pt.bg = c("green", "blue")
  , pch = c(21,22))

# add confidence ellipses
ordiellipse(comm.bc.mds, metadata$Morphotype, conf = 0.95, label =
 TRUE)
ordiellipse(comm.bc.mds, metadata$ SamplingSite, conf = 0.95, label
 = TRUE)

###
```
