# Supplementary material for: Integrating morphology and metagenomics to understand taxonomic variability of Amphisorus (Foraminifera, Miliolida) from Western Australia and Indonesia
Source: PLoS One. 2021 Jan 4;16(1):e0244616. doi: 10.1371/journal.pone.0244616 (PMC7781389; doi:10.1371/journal.pone.0244616)
Supplement: S3 File — (PDF) [file pone.0244616.s005.pdf]

Integrating morphology and metagenomics to understand taxonomic variability of Amphisorus (Foraminifera, Miliolida) from Western Australia and Indonesia

Authors: Jan-Niklas Macher, Martina Prazeres, Sarah Taudien, Jamaluddin Jompa, Aleksey Sadekov, Willem Renema

Supplementary table S1: Sampling site coordinates

Sampling site	latitude	longitude
Pulau Karanrang (SpS specimens)	4°51'32.1"S	119°22'49.3"E
Makasar reef (SpL specimens)	4°52'36.5"S	118°58'50.6"E
Bush Bay	25°11'09.2"S	113°48'52.6"E
Whale Road Beach	26°07'50.1"S	113°38'21.4"E
Horrocks	28°22'18.5"S	114°25'18.8"E
Jurien Bay	30°17'51.9"S	115°01'56.3"E
Parkers Point	32°00'42.7"S	115°32'45.1"E

Supplementary table S2: No. of raw reads and reads retained after quality control and filtering, sampling site and morphotype, shown per specimen

Sample	Total no. of raw reads	Reads after qc	Reads retained after qc (%)	Sampling site	Morphotype
**BB4**	39267732	21661037	55.16243464	Bush Bay	WAL
**BB5**	28686832	15243110	53.13626126	Bush Bay	WAL
**BB10**	15977058	9391725	58.7825681	Bush Bay	WAS
**H1**	28289294	15323297	54.16641716	Horrocks	WAS
**H2**	16012380	8867666	55.38006218	Horrocks	WAS
**H3**	70594132	38329516	54.29561199	Horrocks	WAS
**H4**	16507450	9041172	54.77025222	Horrocks	WAS
**H5**	29735994	15771216	53.03746026	Horrocks	WAS
**H6**	5803252	3095930	53.34819167	Horrocks	WAS
**H7**	14366136	5106932	35.54840355	Horrocks	WAS
**H8**	18640274	10072175	54.03447932	Horrocks	WAS
**H9**	21306486	11413362	53.56754746	Horrocks	WAS
**H10**	24841616	12634510	50.86025804	Horrocks	WAS
**IL2**	14131648	7021495	49.68631401	Pulau Karanrang	SpS
**IL6**	9242340	4741072	51.29731215	Makasar reef	SpL
**IL7**	26467668	14640717	55.31547774	Makasar reef	SpL
**IL9**	17501026	9248511	52.84553603	Makasar reef	SpL
**JB-3**	15075916	8047678	53.38102176	Jurien Bay	WAS
**JB-4**	25068526	14003929	55.86259439	Jurien Bay	WAS
**JB-5**	31166762	16704829	53.59821787	Jurien Bay	WAS
**JB-6**	11694396	6483898	55.44448811	Jurien Bay	WAS
**JB-8**	15215382	7787390	51.18103509	Jurien Bay	WAL
**JB-9**	11625720	6082301	52.3176285	Jurien Bay	WAS
**JB-10**	19092120	10505653	55.02612072	Jurien Bay	WAS
**PP3**	31981440	17753254	55.5111152	Parkers Point	WAS
**PP4**	23095408	12542912	54.30911634	Parkers Point	WAS
**PP5**	22651486	12301738	54.30874601	Parkers Point	WAS
**PP6**	18891972	10272779	54.37642508	Parkers Point	WAS
**PP7**	61163504	34169793	55.86631041	Parkers Point	WAS
**PP9**	17982304	10413124	57.90761851	Parkers Point	WAS
**WRB-1**	63139086	36659983	58.06226432	Whale Road Beach	WAL
**WRB-3**	13223998	7153554	54.09524412	Whale Road Beach	WAL
**WRB-4**	12250654	5935898	48.45372337	Whale Road Beach	WAL
**WRB-5**	15513012	8546981	55.09556107	Whale Road Beach	WAL
**WRB-7**	12876656	6846738	53.17170856	Whale Road Beach	WAL
**WRB-9**	38413570	21266083	55.36086076	Whale Road Beach	WAL
**WRB-11**	14644882	8587852	58.64063637	Whale Road Beach	WAL
**WRB-12**	37753614	21472261	56.87471668	Whale Road Beach	WAL
**Negative_control**	110532	53693	56.87471668		
**% reads in negative control**	0.012146343	0.010842783			
**Total**	910002258	495195764	56.87471668		

Supplementary table S3: Coverage of the 4,305 base pair fragment spanning the 18S- 5.8S- and 28S rRNAs of *Amphisorus* , per sample

Sample	Coverage
**IL2**	54.2
**IL6**	119.2
**IL7**	883.1
**IL9**	217
**BB4**	681.6
**BB5**	315.6
**BB10**	78.1
**WRB1**	992.9
**WRB3**	63.1
**WRB4**	262.4
**WRB5**	183.5
**WRB7**	33.9
**WRB9**	556.3
**WRB11**	371.4
**WRB12**	1611.5
**H1**	406.7
**H2**	52.4
**H3**	564.9
**H4**	197.6
**H5**	564.1
**H6**	101.9
**H7**	263.1
**H8**	361.1
**H9**	336.3
**H10**	341.1
**JB3**	264.5
**JB4**	291.4
**JB5**	266.5
**JB6**	199.7
**JB8**	137.9
**JB9**	72.3
**JB10**	38.1
**PP3**	317.4
**PP4**	95.7
**PP5**	136.1
**PP6**	118.1
**PP7**	408.3
**PP9**	204.2
**Average**	320.0842105

Supplementary table S4: Coverage of the 606 bp 28S rRNA fragment recovered for *Fugacium* Fr5, per sample

Sample	Coverage
BB10	47.8
BB4	82.8
BB5	132.2
H1	282.3
H10	128.7
H2	158.9
H3	606.9
H4	215.6
H5	374.4
H6	59.7
H7	93.8
H8	203.4
H9	225.2
IL6	33.7
IL9	32.9
JB10	195.3
JB3	233
JB4	262.3
JB5	206.7
JB6	113.1
JB8	115
JB9	142.2
PP3	287.8
PP4	76.5
PP5	226.6
PP6	149
PP7	559.4
PP9	193.8
WRB1	219.4
WRB11	268.6
WRB12	187.7
WRB3	10.4
WRB4	32.5
WRB5	64
WRB7	65.1
WRB9	380.1
**Average**	185.1888889

Supplementary table S5: Metagenomic assembled genomes obtained from Amphisorus specimens from Western Australia. MAG completeness, redundancy, taxonomy and abundance (as provided by anvio) are shown.

MAG	MAG completeness	MAG redundancy	Domain	Phylum	Class	Order
**MAG_00001**	100.00%	0.00%	d__Bacteria	p__Proteobacteria	c__Alphaproteobacteria	o__Rhizobiales
**MAG_00002**	100.00%	0.00%	d__Bacteria	p__Proteobacteria	c__Alphaproteobacteria	o__Rhizobiales
**MAG_00003**	100.00%	1.41%	d__Bacteria	p__Proteobacteria	c__Gammaproteobacteria	o__Pseudomonadales
**MAG_00004**	98.59%	0.00%	d__Bacteria	p__Proteobacteria	c__Alphaproteobacteria	o__Rhizobiales
**MAG_00005**	98.59%	0.00%	d__Bacteria	p__Proteobacteria	c__Alphaproteobacteria	o__Caulobacterales
**MAG_00006**	97.18%	0.00%	d__Bacteria	p__Actinobacteriota	c__Acidimicrobiia	o__Microtrichales
**MAG_00007**	97.18%	0.00%	d__Bacteria	p__Proteobacteria	c__Gammaproteobacteria	o__Granulosicoccales
**MAG_00008**	98.59%	1.41%	d__Bacteria	p__Actinobacteriota	c__Acidimicrobiia	o__Microtrichales
**MAG_00009**	98.59%	1.41%	d__Bacteria	p__Proteobacteria	c__Alphaproteobacteria	o__Caulobacterales
**MAG_00010**	98.59%	1.41%	d__Bacteria	p__Proteobacteria	c__Alphaproteobacteria	o__Micavibrionales
**MAG_00011**	98.59%	1.41%	d__Bacteria	p__Proteobacteria	c__Gammaproteobacteria	o__Granulosicoccales
**MAG_00012**	98.59%	1.41%	d__Bacteria	p__Proteobacteria	c__Alphaproteobacteria	o__Rhizobiales
**MAG_00013**	98.59%	2.82%	d__Bacteria	p__Proteobacteria	c__Gammaproteobacteria	o__UBA4486
**MAG_00014**	97.18%	1.41%	d__Bacteria	p__Bacteroidota	c__Bacteroidia	o__Flavobacteriales
**MAG_00015**	95.77%	0.00%	d__Bacteria	p__Bacteroidota	c__Bacteroidia	o__Flavobacteriales
**MAG_00016**	97.18%	1.41%	d__Bacteria	p__Proteobacteria	c__Gammaproteobacteria	o__Granulosicoccales
**MAG_00017**	97.18%	1.41%	d__Bacteria	p__Proteobacteria	c__Gammaproteobacteria	o__Granulosicoccales
**MAG_00018**	97.18%	1.41%	d__Bacteria	p__Proteobacteria	c__Alphaproteobacteria	o__Micavibrionales
**MAG_00019**	98.59%	2.82%	d__Bacteria	p__Proteobacteria	c__Alphaproteobacteria	o__Rhodobacterales
**MAG_00020**	92.96%	1.41%	d__Bacteria	p__Bacteroidota	c__Bacteroidia	o__Cytophagales
**MAG_00021**	95.77%	4.23%	d__Bacteria	p__Bacteroidota	c__Bacteroidia	o__Flavobacteriales
**MAG_00022**	92.96%	1.41%	d__Bacteria	p__Proteobacteria	c__Gammaproteobacteria	o__UBA12402
**MAG_00023**	84.51%	2.82%	d__Bacteria	p__Patescibacteria	c__Saccharimonadia	o__Saccharimonadales
**MAG_00024**	73.24%	4.23%	d__Bacteria	p__Bacteroidota	c__Bacteroidia	o__Flavobacteriales
**MAG_00025**	61.97%	2.82%	d__Bacteria	p__Proteobacteria	c__Alphaproteobacteria	o__Rhodobacterales
**MAG_00026**	56.34%	1.41%	d__Bacteria	p__Proteobacteria	c__Gammaproteobacteria	o__Granulosicoccales

MAG	BB10	BB4	BB5	H1	H10	H2	H3	H4	H5	H6	H7	H8	H9	JB10
**MAG_00001**	0.2699527	0.00663675	0.08312122	0.70386749	0.37786765	46.6847392	0.00360686	0.00929891	0.00611752	0.00993177	0.0021902	0.00518569	0.00267392	0.00612981
**MAG_00002**	0.46854551	0.00859082	0.14460009	2.86979452	2.98861903	0.08100829	1.49144689	8.52757565	7.54349043	1.17627978	0.42749731	0.04354483	0.10014899	0.00087907
**MAG_00003**	0.00075373	0.00020021	0.00147325	0.00017798	0	2.07E-05	9.95E-05	0.00028975	0.00018909	0.00088813	0.00021575	0.00036812	0	10.0947626
**MAG_00004**	0.05529979	0.41059756	0.01248678	0.00108681	0.00082962	0.00040555	0.00098112	0.0013155	0.00064705	0.00154614	0.00264623	0.00064325	0.00124568	0.00031415
**MAG_00005**	0.55304062	0.00087929	0.00220701	0.0005035	0.00017124	0.0001062	0.00033041	0.00080302	0.00059714	0.00107069	0.00021069	0	3.22E-05	6.81E-05
**MAG_00006**	0.28171428	4.76949197	3.46218781	2.47566088	0.76418274	1.28751312	0.68721255	0.91958775	1.40232358	3.20815342	5.86879249	1.75359751	0.99311578	0.0008958
**MAG_00007**	3.61501256	1.30543667	16.9632251	0.00580317	0.00687237	0.00435491	0.0070455	0.00474029	0.00549024	0.01621343	0.02206273	0.01689284	0.00679639	0.00446685
**MAG_00008**	0.49161809	9.12182533	6.62029962	1.17138842	0.38023979	0.65970581	0.35390779	0.45641586	0.68433879	1.67618538	2.8361922	0.95159151	0.55334384	0.00070884
**MAG_00009**	1.609009	0.00510919	0.00879303	5.09E-05	0.00013015	0.00012105	9.58E-05	3.61E-05	0	0.00087919	0.00042375	0.00038063	0.00039846	0.00013029
**MAG_00010**	0.08990641	0.40512	0.31911218	0.0967424	0.01821576	0.29613865	0.00136368	0.22165796	0.00485082	0.98286737	0.03653145	1.08003111	0.80320864	0.00023236
**MAG_00011**	1.9893694	0.98062693	0.65569835	2.07223365	1.79578349	1.1394219	2.94200547	1.59599203	1.69712743	4.06164728	5.72401288	2.16066392	1.88233105	1.051035
**MAG_00012**	0.08898545	0.23830734	0.02445808	0.00123287	0.00058448	0.00050397	0.0006549	0.00114947	0.00089206	0.00098647	0.00032355	0.00057527	0.00128705	0.00049886
**MAG_00013**	8.17E-05	0	0.00021695	3.68E-05	0	0.00019074	0.00021673	0.00012802	0.00053773	0.00346915	0	0.00023704	0	9.5123523
**MAG_00014**	0.01601438	0.00100972	0.00603262	0.0039481	0.00535124	5.67016832	0.87585054	0.13658666	0.00271466	0.01476894	2.91839206	3.44142194	0.66758934	0.00058586
**MAG_00015**	2.65066401	0.90027161	2.93586039	2.06813825	0.51501248	0.00579816	0.20471356	0.78127222	0.4388395	3.90409414	0.17070385	0.79810518	1.3438811	0.00857669
**MAG_00016**	3.12482452	2.38786916	1.59428375	0.93501104	0.79170842	0.48478859	1.23387534	0.71658257	0.75940727	1.91504957	2.63686734	0.94970227	0.85303457	0.48612033
**MAG_00017**	1.97654599	0.00420145	0.0108088	0.00266818	0.00198162	0.00323636	0.00434672	0.00550917	0.00274326	0.10838433	0.00846936	0.00301047	0.05910466	0.01875898
**MAG_00018**	0.04019035	0.17300534	0.12550136	0.19153232	0.02109654	0.79156791	0.0023688	0.32991523	0.00499761	2.23879933	0.04837332	2.99027341	1.87021615	0.00027365
**MAG_00019**	0.44569925	0.00268532	0.00599869	0.00156071	0.00227029	0.00057852	0.00109153	0.00384818	0.00143159	0.00441713	0.00115952	0.00422069	0.00308127	0.00286598
**MAG_00020**	0.43314269	0.00305277	0.00063594	0.00012204	6.15E-05	0.00125673	1.92E-05	0.00021478	0.00055726	8.21E-05	0.00058815	0.10850006	0.00215778	0.00041841
**MAG_00021**	0.00976996	0.2052479	0.00481379	0.00040358	0.00032944	0.00107975	0.00114133	0.00401224	0.00046437	0.01243795	0.00283348	0.00632058	0.12265364	21.2055633
**MAG_00022**	0.00024955	0	0	0.00037929	0	0	0	0	8.47E-05	0	0	0	0	0
**MAG_00023**	0.07760571	0.13868697	0.10222342	1.20038476	0.02127524	9.17310614	0.00048264	0.92317111	0.00662469	0.00893852	1.11451203	0.43679617	0.00104955	0
**MAG_00024**	0.40146585	0.01033381	0.7238327	0.02044964	0.01336952	0.0034688	0.15962094	0.0085518	0.00442261	2.3694582	0.84704992	1.89600124	0.08903677	0.95929306
**MAG_00025**	0.61636293	0.00143096	0.0570699	9.98E-05	0.00014363	0	0.00011427	0.00050148	0	0.00053675	0.00023961	0.00030454	0.00050436	0.00010225
**MAG_00026**	0.06201748	0.01712731	0.15831713	0.00066546	0	0	0.00056679	0	0.00031721	0.00111563	0.00095754	0	0.00047288	0

MAG	JB3	JB4	JB5	JB6	JB8	JB9	PP3	PP4	PP5	PP6	PP7
**MAG_00001**	0.00755156	0.00447847	0.00254644	0.00293152	0.00780417	0.00402241	0.00842265	0.64075736	0.00201141	0.03142194	0.00254022
**MAG_00002**	0.0005516	0.00048276	0.00030302	0.00020065	0.00033633	0.00113413	0.03382691	0.00429235	0.0002129	0.03046681	0.00065877
**MAG_00003**	0.00031026	0.00012387	0.0003842	0.00021573	0.00217537	0.00170996	0.00010812	0	0	0.00019103	4.19E-05
**MAG_00004**	0.00017474	0.00132744	0.0010399	0.00065917	0.001327	0.00019175	2.52206307	0.0084093	0.00261871	0.03123386	0.00150467
**MAG_00005**	0.00036754	0.00061327	0.00056392	0.00056982	0.00012732	0.0001577	0.00049146	0.02170116	0.00063979	0.21857233	0.00027971
**MAG_00006**	0.00195289	0.00600999	0.01331043	0.00083526	0.00945039	0.00155201	0.1193368	0.19569291	0.00823095	0.57298312	1.35452521
**MAG_00007**	0.00110863	0.00924242	0.01332625	0.00670446	0.0023999	0.00303163	0.00281415	0.13798794	0.01171682	1.21271534	0.00325289
**MAG_00008**	0.00216046	0.00357561	0.00890902	0.00078286	0.00674041	0.0012096	0.07416616	0.14414055	0.00720174	0.52047257	0.79453576
**MAG_00009**	0.00037547	0.01197369	0.00096991	0.00047667	0.00071905	0.00054524	0.0005545	0.06111234	0.00130366	0.38597502	0.00060344
**MAG_00010**	0.00141958	0.00060756	0.00086937	0.00103375	0.00048814	0.00097261	0.00059262	0.00654809	0.05392638	0.03588507	0.00071828
**MAG_00011**	0.2622665	1.81290376	0.23839713	1.28699899	0.0253991	0.26827447	0.09576362	0.98910315	0.2521075	2.80791429	0.01322378
**MAG_00012**	0.00127611	0.00085487	0.00040695	0.00141051	0.00063452	0.00056833	6.83467382	0.00571675	0.00138291	0.02143294	0.00135928
**MAG_00013**	0.00029213	0.00025073	0.00020572	0	0.00026458	0.00046425	3.56E-05	0	0	3.34E-05	8.75E-06
**MAG_00014**	0.00404895	0.00261651	0.00184569	0.00396025	0.00079677	0.03162348	0.00190884	0.00710218	0.00071208	0.00915859	0.00540043
**MAG_00015**	0.07159933	0.30655246	1.64738128	4.16391886	0.47270948	0.43164798	0.6600099	2.3610546	0.02397483	3.63776724	4.00666762
**MAG_00016**	0.11814152	0.84670139	0.1168081	0.55075381	0.01636203	0.13079045	0.05309774	0.56153847	0.1379584	1.73064396	0.01572347
**MAG_00017**	0.38245259	0.13492873	3.90590149	0.84129871	2.62909727	0.76207499	2.59431955	0.89894095	2.28148934	3.13591301	4.09638765
**MAG_00018**	0.00027455	9.41E-05	0.00090029	8.74E-05	0	0	9.92E-05	0.00663812	0.07481982	0.03357046	4.53E-05
**MAG_00019**	0.00427893	0.0015432	0.00246659	0.06517251	0.00282262	0.0009166	0.03793433	2.21230304	0.53702303	2.00815761	1.98346261
**MAG_00020**	1.68414648	1.6562002	0.09401636	1.29009025	0.68526993	0.01706898	0.79927472	0.51878291	0.16420386	0.17081653	0.70227802
**MAG_00021**	0.00051776	0.00328838	0.00199305	0.00134149	0.00381917	0.00338252	0.00216294	0.00854267	0.00992663	0.00561614	0.00057138
**MAG_00022**	0.00057726	8.67E-05	0	0.0005248	0	0	0.00071937	0.00108359	0	0.00022421	0.00020918
**MAG_00023**	0	0.00056641	0.00026187	0.00034672	0.00022611	0.00023885	0.00019616	0	0	0.00574376	0.00020097
**MAG_00024**	0.00279695	0.16436944	0.33605481	0.04557489	1.3828383	0.28421582	0.03641828	2.13914698	0.25302795	0.12997983	0.01025472
**MAG_00025**	0.00071206	0.00051481	0.00014496	0.00052688	0.00054639	0.00013468	1.39335737	0.00342304	0.0067198	0.03484167	0.00069955
**MAG_00026**	0	0.00022701	0	0	0	0	0.00041889	0.00423275	0.00121337	0.02420304	0.00113825

MAG	PP9	WRB1	WRB11	WRB12	WRB3	WRB4	WRB5	WRB7	WRB9
**MAG_00001**	0.00241338	0.00507465	0.00934691	0.00318547	0.18224044	0.02123459	0.00649937	0.00516132	0.00939427
**MAG_00002**	0.00011342	0.02203065	2.49044231	0.0516789	2.1976016	0.36103821	0.0115068	0.02186176	0.02356532
**MAG_00003**	0.00016593	0.00128099	0.00066357	0.00130257	0.00371579	0.0007978	0.0005177	0.00157203	0.00223431
**MAG_00004**	0.00111838	0.00422544	0.94836868	0.05232619	1.4890153	5.52356178	0.05212755	0.00851003	4.84902297
**MAG_00005**	0.00018488	0.00122685	0.00086895	0.00123164	24.3977278	0.00971506	0.09569738	0.00817927	0.00293433
**MAG_00006**	0.54720621	0.19274899	0.01033767	4.79801625	2.03966568	2.09830614	0.55450085	0.02217543	0.01860751
**MAG_00007**	0.02088511	21.9238206	5.26772326	16.8404658	23.032816	10.1540179	6.41299278	12.3113248	18.8593487
**MAG_00008**	0.33972331	0.47779818	0.02115688	11.9529328	5.09749549	5.35313137	1.40267968	0.06147717	0.04833905
**MAG_00009**	0.00045238	0.01324602	0.00235275	0.00806096	34.2749972	0.05412859	0.13197477	0.0212137	0.01734382
**MAG_00010**	0.10288714	2.84558504	0.35359552	3.10512877	0.52568806	1.83989989	1.00830225	0.67771843	0.67177742
**MAG_00011**	1.88152469	0.99076152	1.63676905	2.09436531	11.0765471	0.48334586	1.14224546	1.31388494	1.01739338
**MAG_00012**	0.0007639	0.00172432	0.46723063	0.02708786	0.54873059	1.94996341	0.02291749	0.00209938	2.09379424
**MAG_00013**	0	3.19E-05	0	0	6.96E-05	0.00064824	0	0	0
**MAG_00014**	0.00159853	0.00098242	0.00066993	0.00122024	0.00296348	0.00215761	0.00270843	0.00318606	0.0013425
**MAG_00015**	1.09351605	0.04159671	0.00422147	0.02329678	0.11346642	0.42064195	0.10124985	0.07998632	0.04215897
**MAG_00016**	0.95814604	2.53664581	4.26333638	5.56019927	31.1453657	1.29278595	2.89685702	3.52629747	2.78879562
**MAG_00017**	0.02527506	0.00714672	0.00443854	0.00558902	0.04879006	0.00182562	0.00557347	0.07909539	0.02397651
**MAG_00018**	0.14500828	0.96922009	0.15840734	1.07050846	0.19225697	0.686097	0.38106945	0.21945088	0.25219912
**MAG_00019**	0.54092906	0.44015718	0.00642513	0.00695537	1.10255643	0.02375073	0.00860735	0.02049019	0.00783422
**MAG_00020**	0.00670218	0.38296699	0.17692749	0.0313614	0.02033165	0.06364128	0.00111252	0.00971966	0.00068649
**MAG_00021**	0.00047965	0.00678456	0.00103206	0.01628264	0.00592997	0.00744912	0.00867948	0.01164289	0.03921709
**MAG_00022**	0	0.55657892	0.00317827	0	0.14563843	0.49337609	33.0838181	0.00101189	0.00122077
**MAG_00023**	0.00120522	0.02533457	0.00165602	1.76255527	0.52157043	1.22468169	0.20131076	0.0016799	0.00040367
**MAG_00024**	0.0112365	0.62905685	0.03107354	0.00335466	0.05021209	0.15998089	1.0329464	1.55712092	0.07813586
**MAG_00025**	0.00013673	0.00643545	0.0047632	0.00386736	2.7438535	8.45487597	0.04272975	0.05428141	0.91364148
**MAG_00026**	0.00515095	0.35860383	0.09740336	0.36113304	0.34555656	0.17685228	0.64636615	0.21983345	27.7786399
